# Supplementary material for: Asynchronous magnetic resonance elastography: Shear wave speed reconstruction using noise correlation of incoherent waves
Source: Magn Reson Med. 2022 Oct 27;89(3):990–1001. doi: 10.1002/mrm.29502 (PMC9792433; doi:10.1002/mrm.29502)
Supplement: Supplementary file 1 — DATA S1 MATLAB code used to generate the numerical simulations of Figure 1 [file MRM-89-990-s002.zip › k-Wave/helpfiles/example_pr_3D_fft_planar_sensor.html]

3D FFT Reconstruction For A Planar Sensor Example (k-Wave)


# 3D FFT Reconstruction For A Planar Sensor Example

This example demonstrates the use of k-Wave for the reconstruction of a three-dimensional photoacoustic wave-field recorded over a planar array of sensor elements. The sensor data is simulated using `kspaceFirstOrder3D` and reconstructed using `kspacePlaneRecon`. It builds on the Simulations In Three Dimensions and 2D FFT Reconstruction For A Line Sensor examples.

- Open the file in the MATLAB Editor
- Run the file in MATLAB

## Contents

- Simulating the sensor data
- Performing the reconstruction

## Simulating the sensor data

The sensor data is simulated using `kspaceFirstOrder3D` in the same way as in the preceding simulation examples. The initial pressure is created using `makeBall` and is again smoothed prior to invoking the simulation. The sensor mask is defined to be a planar sensor array (over a plane x = const). A visualisation of the initial pressure distribution and the sensor mask using `voxelPlot` is given below.

```
% smooth the initial pressure distribution and restore the magnitude
source.p0 = smooth(p0, true);

% define a binary planar sensor
sensor.mask = zeros(kgrid.Nx, kgrid.Ny, kgrid.Nz);
sensor.mask(1, :, :) = 1;
```


## Performing the reconstruction

The reconstruction is invoked by calling `kspacePlaneRecon` with the sensor data (recorded over a planar sensor array), as well as the properties of the acoustic medium and the sampling parameters. By default, the sensor data input must be indexed as `p_tyz(time, sensor_y_position, sensor_z_position)`. Alternatively, the optional input parameter `'DataOrder'` can be set to `'yzt'` (the default setting is `'tyz'`). In both cases, the simulated `sensor_data` returned by `kspaceFirstOrder3D` must first be reshaped.

```
% reshape sensor data to y, z, t
sensor_data_rs = reshape(sensor_data, Ny, Nz, kgrid.Nt);

% reconstruct the initial pressure
p_xyz = kspacePlaneRecon(sensor_data_rs, kgrid.dy, kgrid.dz, kgrid.dt, ...
    medium.sound_speed, 'DataOrder', 'yzt', 'PosCond', true, 'Plot', true);
```

The size of the recorded data and the time to compute the reconstruction are both printed to the command line.

```
Running k-Wave planar reconstruction...
  grid size: 212 by 44 by 44 grid points
  interpolation mode: *nearest
  applying positivity condition...
  computation completed in 0.14516s
```

A plot of the initial pressure distribution and the reconstructed initial pressure distribution with a positivity condition (interpolated onto a k-space grid with the same dimensions) are shown below. The three displayed image planes intersect at the centre of ball shown above. An animated slice-by-slice visualisation of the reconstructed initial pressure distribution can also be viewed using `flyThrough`.
